# Mutation in Bruton Tyrosine Kinase (BTK) A428D confers resistance To BTK-degrader therapy in chronic lymphocytic leukemia

**DOI:** 10.1038/s41375-024-02317-4

**Published:** 2024-07-24

**Authors:** Richard L. Wong, Michael Y. Choi, Huan-You Wang, Thomas J. Kipps

**Affiliations:** 1 Division of Laboratory and Genomic Medicine, Department of Pathology, University of California San Diego, La Jolla, CA USA; 2 Division of Hematology/Oncology, Department of Medicine, Moores Cancer Center, UC San Diego, La Jolla, CA 92093 USA; 3 Center for Novel Therapeutics, Division of Hematology/Oncology, Department of Medicine, Moores Cancer Center, UC San Diego, La Jolla, CA 92093 USA

**Keywords:** Chronic lymphocytic leukaemia, Genetics research

## Abstract

Targeting BTK has profoundly changed the face of CLL treatment over the past decade. Iterative advances in the cat and mouse game of resistance and redesign have moved BTK inhibitors from covalent to non-covalent and now targeted protein degraders. However, contrary to the presumption that protein degraders may be impervious to mutations in BTK, we now present clinical evidence that a mutation in the kinase domain of BTK, namely A428D, can confer disease resistance to a BTK degrader currently in clinical trials, that is BGB-16673. Modeling of a BTK A428D mutation places a negatively charged aspartic acid in place of the hydrophobic side chain of alanine within the binding pocket of another BTK-degrader in clinical development, namely NX-2127, suggesting that CLL cells with BTK A428D also may be resistant to NX-2127, as they already are known to be with either non-covalent or covalent inhibitors of BTK. Consequently, the two BTK degraders furthest advanced in clinical trials potentially may select for CLL cells with BTK A428D that are resistant to all approved BTKi’s.

## To the Editor:

The enzyme Bruton tyrosine kinase (BTK) is involved in B-cell signaling that promotes the migration, proliferation, and survival of neoplastic B-cells of patients with chronic lymphocytic leukemia (CLL) [1]. Consequently, drugs that block the action of BTK are highly effective and fundamentally have changed the standard of care for patients with CLL since the therapeutic introduction of the first BTK inhibitor (BTKi), ibrutinib, in 2013. Ibrutinib, and second-generation BTKi’s, such as acalabrutinib and zanubrutinib, form a covalent bond with BTK within its kinase domain at residue C481, causing permanent inactivation of BTK and requiring the leukemia cell to synthesize new enzyme to restore B-cell signaling [2]. A nonsynonymous mutation in BTK at C481, such as C481S, can block the capacity of these drugs to form a covalent bond with BTK, thus conferring clinical resistance to ‘covalent’ BTKi’s [3]. This stimulated development of ‘non-covalent’ BTKi’s, such as pirtobrutinib or nemtabrutinib, which may be effective in treating patients with CLL cells that harbor the BTK C481S mutation [4]. However, patients subsequently were noted to develop resistance to therapy with pirtobrutinib through acquisition of other nonsynonymous BTK mutations, e.g. T474I or L528W [5]. Furthermore, other BTK mutations that confer clinical resistance to covalent and/or noncovalent BTKi’s now have been identified, including V416L, A428D, and M437R [6–8]. This has stimulated interest in development of drugs that can target BTK for proteasomal degradation, the so-called “BTK-degraders” [9], which are presumed able to mitigate the risk of drug resistance due to mutations in BTK [10].

Here we describe a patient who successively acquired resistance to each generation of BTKi, including a BTK-degrader, BGB-16673, which has demonstrated clinical activity in early phase I clinical trials [11]. He was 66 years old when diagnosed in 2014 with CLL that expressed an unmutated IGHV with 100% homology to IGHV1-69 and that reportedly had trisomy 12 as the sole cytogenetic abnormality detected by Fluorescence in situ Hybridization (FISH). Due to rapid disease progression with a lymphocyte-doubling time of ≈4 months, he was treated with bendamustine and rituximab in 2015, achieving a clinical complete response (CR) by iwCLL criteria [12]. Due to relapsed progressive CLL in 2017, he presented to us for therapy with obinutuzumab and venetoclax (after debulking with high dose methylprednisolone), again achieving a clinical CR. He remained on therapy with venetoclax until 2019 when he developed rapidly progressive lymph node enlargement, for which he initiated ibrutinib, resulting in complete resolution of his bulky lymphadenopathy. In 2021 he experienced symptomatic cardiac arrythmias, thought possibly ibrutinib-related; consequently his therapy was switched to acalabrutinib. He maintained a good clinical response until 14 months later, when he again developed progressive lymphadenopathy. Next generation sequencing (NGS) of his marrow aspirate, of which 20% were CLL cells (**07/07/2022**, Table 1), revealed a mutation in *BTK* c1442G>C at a variant allelic frequency (VAF) of 3.1%, resulting in BTK p.C481S. Also noted was a mutation in *TP53* c.730G>A at a VAF of 10%, resulting in TP53 p.G244S and a complex karyotype with del(17p) (Table 1). He initiated therapy with an inhibitor of the δ/γ isoforms of phosphoinositide-3-kinase, duvelisib, which again resulted in complete resolution of his lymphadenopathy. However, because of adverse effects of therapy, he discontinued duvelisib and initiated therapy with pirtobrutinib. His disease was well controlled on pirtobrutinib for 7 months, after which time he again developed rapidly progressive lymphadenopathy. NGS of his marrow aspirate, of which 42% were CLL cells (**09/12/2023**, Table 1), revealed a distinctive subclone of CLL harboring a mutation in *BTK* c.1421C>T at a VAF of 19%. This mutation resulted in BTK p.T474I, which prior studies found confers resistance to pirtobrutinib [6]. Also detected was the previously identified mutation in *TP53* c.730G>A at a VAF of 35% and similar complex karyotype, but not the mutation in *BTK* c1442G>C (Table 1). After this analysis, he discontinued pirtobrutinib and enrolled into a clinical study, in which he was treated with the BTK-degrader, BGB-16673. After initially experiencing a marked reduction in bulky lymphadenopathy, he came off this study after 4 months of therapy because of rapidly progressive disease. Subsequent to his coming off-study, NGS of his marrow aspirate, of which 67% were CLL cells (**02/15/2024**, Table 1), revealed yet another distinctive subclone of CLL cells with a new mutation in *BTK* c.1283C>A at a VAF of 28%, resulting in BTK p.A428D, but without detectable *BTK* c1442G>C or *BTK* c.1421C>T, while maintaining the same mutation in *TP53*, namely c.730G>A, at a VAF of 62% and same complex karyotype. These findings support a model of selective emergence of distinct subclones during his successive therapy with each type of BTKi from cells harboring the same mutation in *TP53*, namely c.730G>A and complex karyotype as noted in earlier samples. Remarkably there did not appear to be substantial evolution in the karyotypic complexity noted in the cytogenetics or FISH analyses performed on samples collected on 07/22/2019, 07/07/2022, 09/12/2023, and 02/15/2024, which respectively were obtained before and after treatment with obinutuzumab, venetoclax, and successive BTKi’s (Table 1 and Supplementary Fig. 1 and Supplementary Table 1).  In any event, this case highlights the challenges of targeting BTK in some patients with CLL.

**Table 1:** The column on the left indicates the date of sample collection.

Date	TP53 c.730 G > A (G244S)	BTK c.1442 G > C (C481S)	BTK c1421C>T (T474I)	BTK c.1283 C > A (A428D)	Cytogenetics
07/22/19	**–**	**–**	**–**	**–**	Abnormal karyotype- multiple complex clones with gain of chromosome 12, loss of 17p and translocation(s) involving 2p?15, 14q32 and 19q13.3. Abnormal FISH result- gain(s) of chromosome 12 (40% of cells), IGH translocation(s) (52–80% of cells) and loss of 17p (47% of cells).
07/07/22	10%	3.1%	N.D.	N.D.	Abnormal karyotype - complex polyploid clone with relative gain of chromosome 12, relative loss of 17p and translocation(s) involving 2p?15, 14q32 and 19q13.3. Abnormal FISH result - polyploidy with gains of chromosome 12, likely IGH translocation(s) and relative loss of 17p in 7–8% of cells.
09/12/23	35%	N.D.	19.3%	N.D.	Abnormal karyotype - complex polyploid clone with relative gain of chromosome 12, relative loss of 17p and translocation(s) involving 2p?15, 14q32 and 19q13.3. Abnormal FISH result - polyploidy with relative gain of chromosome 12 (x5), likely IGH translocation(s) and relative loss of 17p (x2) in 48–58% of cells.
02/15/24	62%	N.D.	N.D.	28%	Abnormal karyotype- complex polyploid clone with relative gain of chromosome 12, relative loss of 17p, and translocation(s) involving 2p?15, 14q32, and 19q13.3. Abnormal FISH result- polyploidy with relative gain of chromosome 12 (x5), likely IGH translocation (s), and relative loss of 17p (x2) in 65–79% of cells.

Each of the next four columns provides the name of the gene, the mutation detected, and the amino acid residue followed by the position number in the encoded protein and then the resulting amino acid substitution using the 20-letter alphabet for amino acid residues. The numbers below provide the variant allelic frequency (VAF) of the specific mutation detected through Illumina sequencing platforms with in-house gene panels and manual review of the data to examine for specific mutations in BTK. The respective read depth for BTK mutations at p.481, p.474, or p.428 was: 600, 541, or 590 (on **07/07/22)**, 927, 830, or 1970 (on **09/12/23)** and 550, 480, or 622 (on **02/15/24)**. A dash (-) indicates that no data are available from that date and N.D. means ‘Not Detected’, which means that the number of observed mutations, if any, at this site was below the threshold considered significant or above background (e.g. VAF ≤ 0.4%). The far-right column summarizes the noted cytogenetics of the sample collected on the specified date. A question mark (?) before a chromosome or structural abnormality indicates that the marker chromosome or structural abnormality cannot be identified unambiguously. Pictures of representative metaphases and detailed cytogenetic findings are provided in Supplementary Fig. 1. Under these cytogenetic results are the results of fluorescence in situ hybridization (FISH) performed on 200 interphase nuclei of CLL cells collected on the date specified, using a panel of probes to detect chromosomal abnormalities commonly associated with CLL: CCND1/IGH for translocation (11;14)(q13;q32), ATM (11q22.3) for deletion 11q, D12Z3 (12 centromere) for trisomy 12, D13S319 (13q14.3) for deletion 13q14.3, LAMP1 (13q34) for deletion of 13q34, and TP53 (17p13.1) for deletion 17p (Abbott Molecular, Inc.). A detailed report of FISH data are provided in Supplementary Table 1.

The BTK A428D mutation is located in the ATP-binding interface of BTK and significantly reduces its capacity to undergo autophosphorylation at Y223 or phosphorylation of its downstream substrate, phosphatidylinositol-specific phospholipase Cγ2 (PLCγ2) [7, 8]. However, upon B-cell receptor ligation the mutant BTK A428D, along with other catalytically inactive BTK mutants, e.g. BTK V416L or L528W, still can enable release of inositol-1-phosphate and provide for an enhanced Ca^2+^ flux in response to anti-µ stimulation in cells that have such mutant BTK when compared with cells with wildtype BTK [6, 8]. As such, these BTK mutant proteins can enable downstream signaling leading to activation of AKT, ERK, and NF-κB upon B-cell receptor ligation that is not inhibited by pirtobrutinib [6, 8, 9, 13]. Testament to this is the finding of the BTK A428D mutation in leukemia cells of patients who develop therapy resistance to pirtobrutinib [6]. Moreover, this mutation also may impair the sensitivity to other non-covalent inhibitors in clinical development, including ARQ-531, fenebrutinib, and vecabrutinib [6], as well as confer resistance to covalent BTKi’s [7]. Nevertheless, the BTK A428D mutation was not detected in the CLL cells of this patient until he developed resistance to the BTK degrader, BGB-16673.

**Fig. 1:**
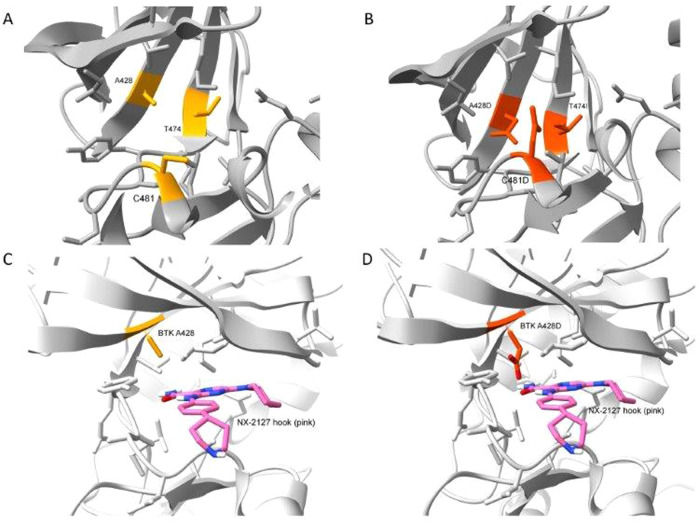
Crystal structures of the BTK kinase domain (PDB ID 8GC7). **A** Crystal structure of wildtype BTK with residues that were found mutated highlighted in yellow. **B** BTK structure highlighting in orange the three modeled resistance mutation variants clustered in the BTK kinase domain. **C** The hook region (in pink) of the BTK degrader NX-2127 bound to wildtype BTK in proximity to the BTK A428 residue (in yellow). **D** Modeled BTK resistance mutation A428D with the aspartic acid side chain (in orange) inside the binding pocket for NX-2127.

This may be due to the selective pressure applied by BTK-degrader therapy. In work presented at the 2023 European Hematology Association Meeting, Feng and colleagues used BGB-16673 to treat various TMD8-derived lymphoma cell lines, each bearing either wildtype BTK or any one of the reported mutated BTK’s that are associated with BTKi resistance (e.g. V416L, A428D, M437R, T474I, T474L, M477I, C481S, C481F, C481Y, or L528W) [14]. They reported that BGB-16673 had a nanomolar IC_50_ and effected BTK protein degradation in each of these TMD8 lymphoma cell lines, except for those with the BTK A428D mutation, suggesting that BGB-16673 interacts with BTK at or around A428. It is noteworthy that the crystal structure of the BTK interacting “hook” of another BTK-degrader in clinical development, namely NX-2127, reveals a close drug interface with the BTK A428 residue [9, 15]. Modeling of a BTK A428D mutation places a negatively charged aspartic acid in place of the hydrophobic side chain of alanine within the binding pocket of NX-2127 to BTK (Fig. 1). This suggests that CLL cells with BTK A428D also may be resistant to NX-2127, as they already are known to be with either non-covalent or covalent BTKi’s [6]. Consequently, the two BTK degraders furthest advanced in clinical trials potentially may select for CLL cells with BTK A428D that are resistant to all approved BTKi’s.

In summary, targeting BTK has profoundly changed the face of CLL treatment over the past decade. Iterative advances in the cat and mouse game of resistance and redesign have moved BTK inhibitors from covalent to non-covalent and now targeted protein degraders. However, contrary to the presumption that protein degraders may be impervious to mutations in BTK, we now present clinical evidence that a mutation in the kinase domain of BTK can confer disease resistance to a BTK degrader currently in clinical trials. Moreover, we anticipate that patients with CLL cells that harbor the A428D mutation in BTK may prove refractory to therapy with either BGB-16673 or NX-2127.

### Supplementary information

Supplementary Figure 1

Legend for Supplementary Figure 1

Supplementary Table 1
