# Supplementary figures and images for: Mutation in Bruton Tyrosine Kinase (BTK) A428D confers resistance To BTK-degrader therapy in chronic lymphocytic leukemia
Source: Leukemia. 2024 Jul 24;38(8):1818–21. doi: 10.1038/s41375-024-02317-4 (PMC11286506; doi:10.1038/s41375-024-02317-4)

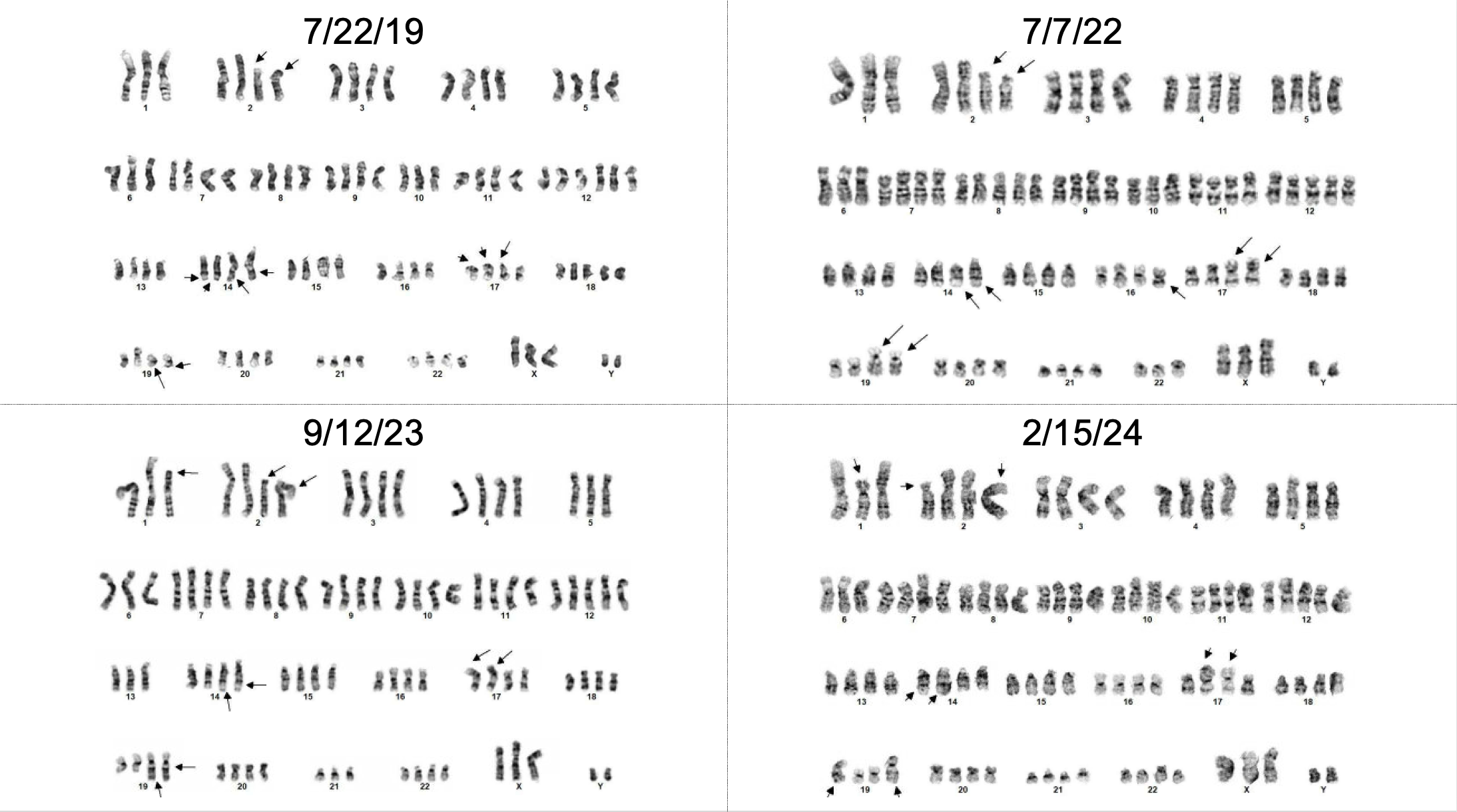

Supplement: Supplementary file 1 — Supplementary Figure 1 [file 41375_2024_2317_MOESM1_ESM.png]
