# Supplementary material for: Mutation in Bruton Tyrosine Kinase (BTK) A428D confers resistance To BTK-degrader therapy in chronic lymphocytic leukemia
Source: Leukemia. 2024 Jul 24;38(8):1818–21. doi: 10.1038/s41375-024-02317-4 (PMC11286506; doi:10.1038/s41375-024-02317-4)
Supplement: Supplementary file 2 — Legend for Supplementary Figure 1 [file 41375_2024_2317_MOESM2_ESM.docx]

**Legend for Supplementary Figure 1**

**7/22/19 -** Representative metaphase chromosomes of CpG-stimulated CLL cells that were collected on 07/22/2019. Twenty-one metaphase cells from CpG-stimulated and unstimulated cultures were examined by G-banding chromosome analysis. Multiple related abnormal clones were identified. Four cells showed clonal abnormalities including a suspected three-way translocation (2;19;14), trisomy 12 and additional material at 17p11.2 resulting in 17p loss. Four cells showed these clonal abnormalities in a tetraploid state along with loss of chromosome 1. One cell showed translocation (2;14), trisomy 12, translocation (14;19) and additional material at 17p11.2 resulting in 17p loss. Two cells showed these abnormalities in a tetraploid state along with loss of chromosomes 1, 6 and 10 and deletion 17p.

47,XY,?t(2;19;14)(p?15;q13.3;q32),+12,add(17)(p11.2)[cp4]/92,idemx2,-1[cp4]/47,XY, t(2;14)(p?15;q32),+12,t(14;19)(q13.3;q32),add(17)(p11.2)[1]/93-94,idemx2,-1,-6,-10,del(17)(p11.2)x2, +mar[cp2]/46,XY[9].

**7/7/22 -** Representative metaphase chromosomes of CpG-stimulated CLL cells that were collected on 07/07/2022. Twenty metaphase cells from CpG-stimulated and unstimulated cultures were examined by G-banding chromosome analysis. Eight polyploid cells showed clonal abnormalities including gain of chromosomes X, 12 and ?20, loss of chromosomes 1, 6, and 10, a suspected three-way translocation (2;19;14), and additional material at 17p11.2 resulting in 17p loss and ?22p11.2.

91~92<4n>,XXYY,+X,-1,?t(2;19;14)(p?15;q13.3;q32)x2,-6,-10,+12,add(17)(p11.2)x2, +?20,?add(22)(p11.2) [cp8]/46,XY[12].

**9/12/23** - Representative metaphase chromosomes of CpG-stimulated CLL cells that were collected on 09/12/2023. Twenty metaphase cells from CpG-stimulated and unstimulated cultures were examined by G-banding chromosome analysis. Eleven near tetraploid cells showed clonal abnormalities including relative gain of chromosomes X and 12, relative loss of chromosome 1, a suspected four-way translocation (1;2;19;14), a suspected three-way translocation (2;19;14), and additional material at 17p11.2 (two copies) resulting in relative loss or 17p.

89~92<4n>,XXYY,+X,-1,?t(1;2;19;14)(p?31;p?15;q13.3;q32),?t(2;19;14)(p?15;q13.3;q32), +12,add(17)(p11.2)x2 [cp11]/46,XY[9].

**2/15/24 -** Representative metaphase chromosomes of CpG-stimulated CLL cells that were collected on 02/15/2024. Twenty metaphase cells from CpG-stimulated and unstimulated cultures were examined by G-banding chromosome analysis. Ten near tetraploid cells showed clonal abnormalities including relative gain of chromosomes X and 12, relative loss of chromosome 1, a suspected four-way translocation (1;2;19;14), a suspected three-way translocation (2;19;14), and additional material at 17p11.2 (two copies) resulting in relative loss or 17p.

90~92<4n>,XXYY,+X,-1,?t(1;2;19;14)(p?31;p?15;q13.3;q32), ?t(2;19;14)(p?15;q13.3;q32), +12,add(17)(p11.2)x2 [10]/46,XY[10].
