# Supplementary material for: Mutation in Bruton Tyrosine Kinase (BTK) A428D confers resistance To BTK-degrader therapy in chronic lymphocytic leukemia
Source: Leukemia. 2024 Jul 24;38(8):1818–21. doi: 10.1038/s41375-024-02317-4 (PMC11286506; doi:10.1038/s41375-024-02317-4)
Supplement: Supplementary file 3 — Supplementary Table 1 [file 41375_2024_2317_MOESM3_ESM.docx]

**Supplementary Table 1**

| **Date** | **FISH Results** | **Interpretation** |
| --- | --- | --- |
| 07/22/19 | nuc ish (ATMx2,TP53x1)[68/200]/(ATMx4,TP53x2)[26/200], (CCND1x2,IGHx3-5)[97/200]/(CCND1x4, IGHx5-10)[63/200], (D12Z3x3-5)[81/200], (D13S319x4)[42/200], (LAMP1x4)[44/200], (3'IGH,5'IGH)x3-5(3'IGH con 5'IGHx2)[23/75]/ (3'IGH,5'IGH)x2-3(3'IGH con 5'IGHx1)[16/75] | Abnormal signal patterns were observed in approximately 50-80% of cells consistent with gain(s) of chromosome 12, probable translocation(s) involving IGH and loss of 17p in a near diploid population and a near tetraploid population. |
| 07/07/22 | nuc ish(CCND1x4)[16/200],(IGHx4~8)[16/200], (ATMx4)[17/200], (D12Z3x5)[17/200], (D13S319x4)[18/200], (LAMP1x4)[13/200], (TP53x2,D17Z1x4)[15/200] | Abnormal signal patterns were observed in approximately 6-9% of cells consistent with polyploidy and gain(s) of chromosome 12, probable translocation(s) involving IGH and loss of 17p. |
| 09/12/23 | nuc ish(ATMx4,TP53x2)[116/200]/(CCND1x4,IGHx5~10)[110/200], (D12Z3x5)[97/200], (D13S319x4)[105/200], (LAMP1x4)[105/200] | Abnormal signal patterns were observed in approximately 48-58% of cells consistent with polyploidy with relative gain of chromosome 12, likely IGH translocation(s) and relative loss of 17p in 48-58% of cells. |
| 02/15/24 | nuc ish(CCND1x4, IGHx5~10)[158/200], (ATMx4,TP53x2)[140/200]/(ATMx3,TP53x2)[16/200], (D12Z3x5)[130/200]/(D12Z3x4)[22/200], (D13S319x4)[152/200], (LAMP1x4)[152/200] | Abnormal signal patterns were observed in approximately 76-79% of cells consistent with polyploidy with relative gain of chromosome 12, likely IGH translocation(s), and relative loss of 17p in 65-79% of cells. |

**Supplementary Table 1 Legend**

The left column provides the date of the sample collection. The middle column provides the FISH data obtained on 200 metaphase nuclei using a panel of probes to detect chromosomal abnormalities commonly associated with CLL: CCND1/IGH for translocation (11;14)(q13;q32), ATM (11q22.3) for deletion 11q, D12Z3 (12 centromere) for trisomy 12, D13S319 (13q14.3) for deletion 13q, LAMP1 (13q34) for deletion of 13q34, and TP53 (17p13.1) for deletion 17p (Abbott Molecular, Inc.). The right column provides interpretation of the FISH data.
